# Identification and validation of exosome-related biomarkers in pediatric glioblastoma

**DOI:** 10.1007/s12672-026-05429-8

**Published:** 2026-06-19

**Authors:** Wenjue Tang, Lingyun Fan, Huihong Dou, Yinlin Tang, Weichun Ma, Huan Peng, Qiongyou Liang, Lifang Nong, Peng Zhang

**Affiliations:** 1 Department of Hematology and Oncology, Children’s Hospital, Maternal and Child Health Hospital of Guangxi Zhuang Autonomous Region, Nanning, 530000 China; 2 Department of Cardiology, Children’s Hospital, Maternal and Child Health Hospital of Guangxi Zhuang Autonomous Region, Nanning, 530000 China; 3 Department of Clinical Laboratory, Children’s Hospital, Maternal and Child Health Hospital of Guangxi Zhuang Autonomous Region, Nanning, China; 4 Guangxi Clinical Research Center for Birth Defects, Guangxi Key Laboratory of Birth Defects Research and Prevention, Guangxi Key Laboratory of Reproductive Health and Birth Defects Prevention, Maternal and Child Health Hospital of Guangxi Zhuang Autonomous Region, Nanning, 530000 China; 5 Medical Genetics and Prenatal Diagnosis Center, Guangxi Academy of Medical Sciences and the People’s Hospital of Guangxi Zhuang Autonomous Region, Nanning, 530000 China

**Keywords:** Pediatric glioblastoma, Exosomes, Biomarkers, FGF9, TGFBR1, PLCB4

## Abstract

Pediatric glioblastoma (pGBM) is an aggressive central nervous system (CNS) tumor whose pathological progression is significantly influenced by exosomal signaling. This study integrated and analyzed transcriptomic datasets (GSE50161, GSE35493) from the Gene Expression Omnibus (GEO), focusing on the intersection between exosome-related genes (ERGs) and disease-associated differentially expressed genes (DEGs). Key biomarkers, FGF9, TGFBR1, and PLCB4, were identified using a machine learning model. The results demonstrated that TGFBR1 expression was up-regulated in the tumor microenvironment, whereas FGF9 and PLCB4 were down-regulated. These genes were closely associated with the γ/α (IFN-γ/α) signaling pathway and E2F target activation. Immune profiling revealed that low expression of FGF9 and PLCB4 correlated with a reduction in central memory CD4^+^T cells, while high TGFBR1 expression was associated with an increase in memory B cells. Further regulatory network analysis uncovered potential epigenetic regulatory mechanisms. In silico drug screening and molecular docking suggested that the histone deacetylase inhibitor Trichostatin A may hold therapeutic potential. This study provides novel biomarkers and insights for the diagnosis and targeted therapy of pGBM.

**Supplementary Information:**

The online version contains supplementary material available at https://doi.org/10.1007/s12672-026-05429-8.

## Introduction

Pediatric glioblastoma (pGBM) is a rare but highly aggressive brain tumor. It makes up about 2.9% of childhood brain cancers [1] and has unique clinical, pathological, and molecular traits. The traditional mode of treatment involving a combination of surgery, radiation, and systemic drugs has not demonstrated an improvement in survival times; less than 20% of children are alive after five years [2]. Patient’s prognosis depends on several factors, including age at diagnosis, where and how the tumor is growing, sex, how much of it the surgeon could remove, its tissue type, and its genetic makeup [3].Presently, the current standard treatment-surgery followed by radiation and the drug temozolomide-fails mostly because resistance develops, or it is hard for these drugs to cross the blood–brain barrier (BBB), leading to frequent recurrences. The biggest weakness of today’s therapies is their inability to effectively target the molecular processes that drive the cancer’s progression [4]. This underscores the urgency of identifying novel biomarkers-such as GPNMB [5, 6], and deciphering core mechanisms like recurrence-linked mesenchymal transition [7]. Progress in these areas is key to unlocking the biological drivers of pGBM and will pave the way for more precise diagnostics and targeted therapie [8, 9] .

Exosomes (Exo) are cell-secreted small vesicles, about 30–150 nanometers in size, that cells release to carry a mix of proteins, lipids, and genetic material like miRNA, lncRNA, mRNA, and DNA. By playing key roles in processes like immunity, tumorigenesis, and therapy resistance, exosomes greatly affect the initiation and progression of many diseases. In glioblastoma, their small size and unique ability to cross the BBB make them promising tools for diagnosis and for predicting how the disease will develop [10, 11]. Exosomes are also released by cancer cells and act as key signaling agents within the tumor microenvironment (TME) [12–14]. In glioblastoma, tumor-derived exosomes have been shown to alter the function of immune cells known as macrophages, creating conditions that support tumor growth, invasion, and immune evasion-ultimately driving disease progression. These findings highlight the potential for developing new therapies aimed at inhibiting or modifying exosomes secreted by glioblastoma stem cells.

Studies showing that exosomes taken from glioma cells treated with drugs can boost signals that promote cell death and lower the levels of heat shock protein. This shows that engineered exosome therapies may have utility for treating cancer [15, 16].

Exosomes are proving to be pivotal in several areas of cancer research, particularly for their potential in delivering drugs directly to tumors, influencing the immune response during immunotherapy, and serving as biomarkers to track glioma progression and how it responds to treatment. While significant strides have been made in understanding exosomes in glioblastoma, our knowledge of their role in the pGBM remains in its early stages. This gap highlights the urgent need for more focused research on exosomes in pGBM. Unlocking their specific functions in this younger patient group could reveal crucial differences in disease development and open new doors for creating more precise diagnostics and powerful treatments tailored to pediatric patients.

This study addresses the major issue of finding an exosome-based biomarker for pGBM diagnosis based on transcriptomic data of the GEO database. We employed a multi-step bioinformatics approach: first identifying potential biomarker candidates linked to exosomes, then conducting functional enrichment and immune infiltration analyses to uncover their biological roles. To understand how these key molecules interact, we constructed molecular regulatory networks. Finally, to explore clinical applications, we combined computer-aided drug prediction with molecular docking simulations. Together, our strategy may constitute a robust approach to building a reliable and multidimensional framework for drug discovery targeting pGBM.

## Results

### The functions and pathways of candidate genes

**Fig. 1:**
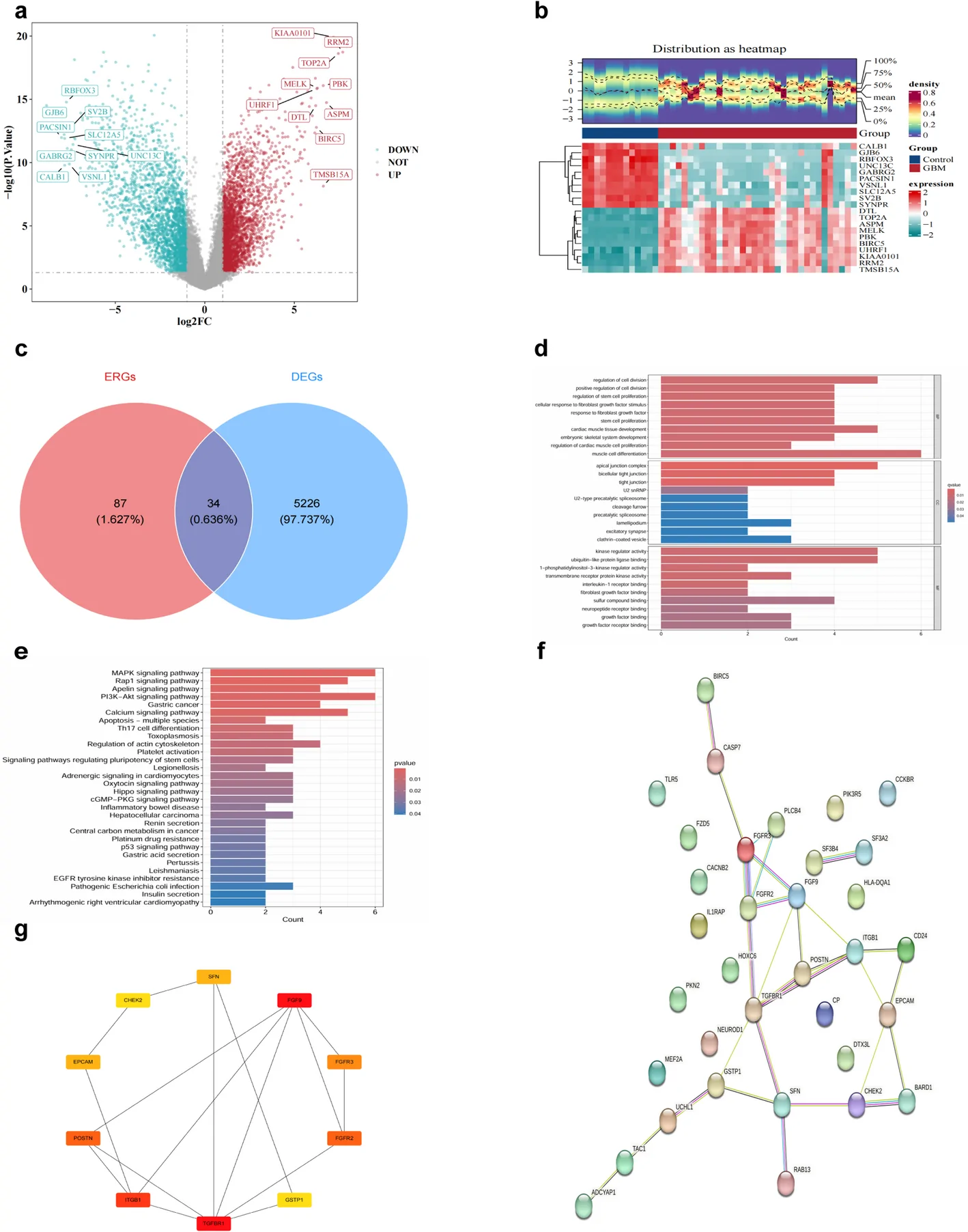
Identification and functions of candidate genes in pGBM. (**a**) Volcanic map of DEGs in pGBM Red: Up-regulated gene, Blue: Down-regulatedgene. Gray: non-significant genes. (**b**) Heat map of DEGs in pGBM. Color code: Orange-red: GBM, Blue: Control. Top: Expression density heatmap (redder: higher density). Bottom: Gene expression heatmap (rows: genes: red: high, green: low. (**c**) Venn diagrams of candidate genes.Blue: DEGs, red: ERG-associated genes, and the overlapping region indicates in both gene sets. (**d**) GO enrichment results of candidate genes. X-axis: Number of enriched genes; Y-axis: GO terms (including BP, CC, and MF). (**e**) KEGG enrichment results of candidate genes. Left: enriched pathway names; Right: number of enriched genes and p-values. Red color intensity scales with p-value magnitude (darker red = larger p-value). (**f**, **g**) PPI network of candidate genes and top 10 genes from MCC algorithm in the network. Nodes: Genes; Edges: Interactions. The darker the color, the higher the algorithm score; genes with robust interactions (including SFN, CHEK2, etc.) are visualized

According to the GSE50161 data analysis, a total of 5260 different genes were differentially expressed between the pGBM and control samples. The GBM group’s expression was up-regulated (*n* = 2800) and down-regulated (*n* = 2460) significantly when compared to the controls (*P* < 0.05; Fig. 1a-b, Table S1). The 34 candidate genes for further analysis (Fig. 1c, Table S2) were discovered via their intersection with 121 ERGs. The investigation of candidate genes for functional enrichment identified 163 significantly enriched Gene Ontology (GO) terms (*P* < 0.05), including 132 biological processes (BPs), 14 cellular components (CCs), and 17 molecular functions (MFs). Among the enriched terms, regulation of cell division, apical junction complex organization and transmembrane receptor protein kinase activity are noteworthy (Fig. 1d, Table S3).

**Fig. 2:**
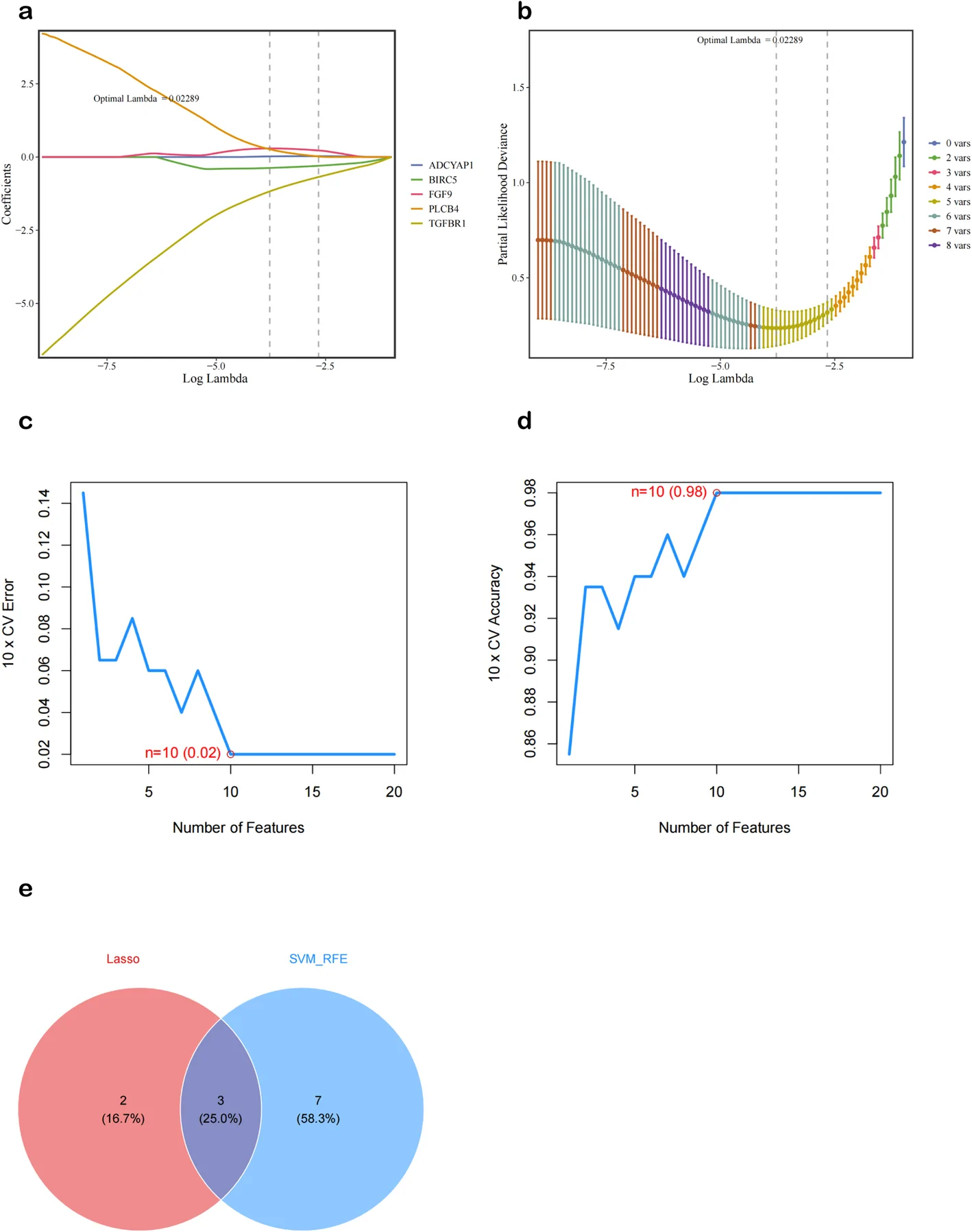
Identified biomarkers for pGBM. (**a**) LASSO (logistic regression) coefficient penalty plot. (**b**) Cross-validation error curve. (**c**) Misclassification rate vs. feature number in SVM-RFE (10-fold CV). X-axis: number of features, Y-axis: 10-fold cross-validation error rate. (**d**) Classification accuracy vs. feature number in SVM-RFE (10-fold CV). X-axis: number of features, Y-axis: 10-fold cross-validation accuracy. (**e**) Venn diagrams of candidate genes. Blue: differentially expressed genes (DEGs), Red: genes selected by Lasso, Blue: genes selected by SVM, Overlap: genes identified by both methods

**Fig. 3:**
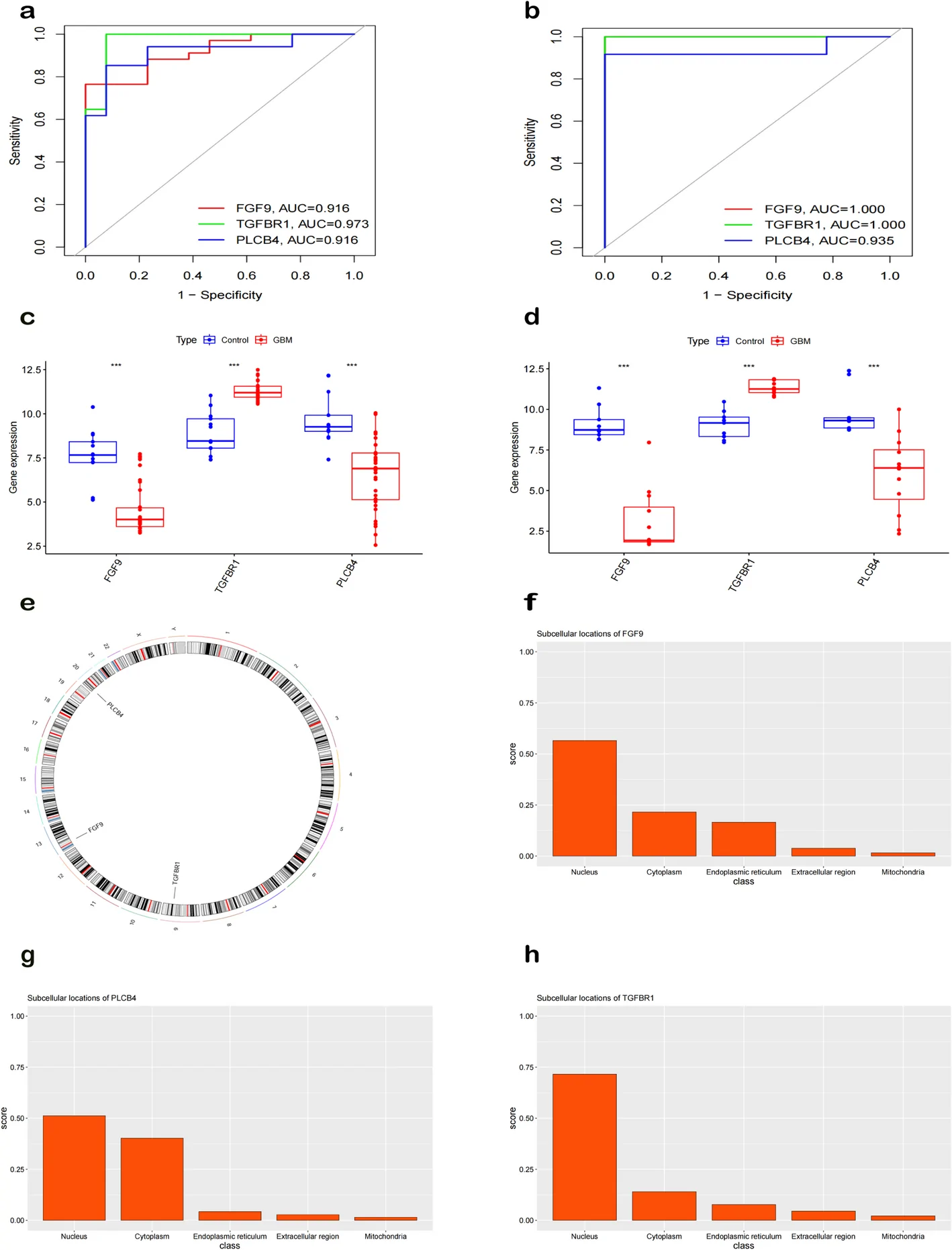
The biomarkers FGF9, TGFBR1, PLCB4. (**a**) ROC: training cohort. (**b**) ROC: validation cohort. (**c**) Expression patterns of candidate key genes in the training set. (**d**) Expression profiles of candidate key genes in the validation cohort. (**e**) Chromosomal localization of candidate genes, Outer ring numbers indicate chromosomes (1–22: human autosomes; XY: sex chromosomes). (**f**–**h**) FGF9, PLCB4, TGFBR1 subcellular localization analysis

**Fig. 4:**
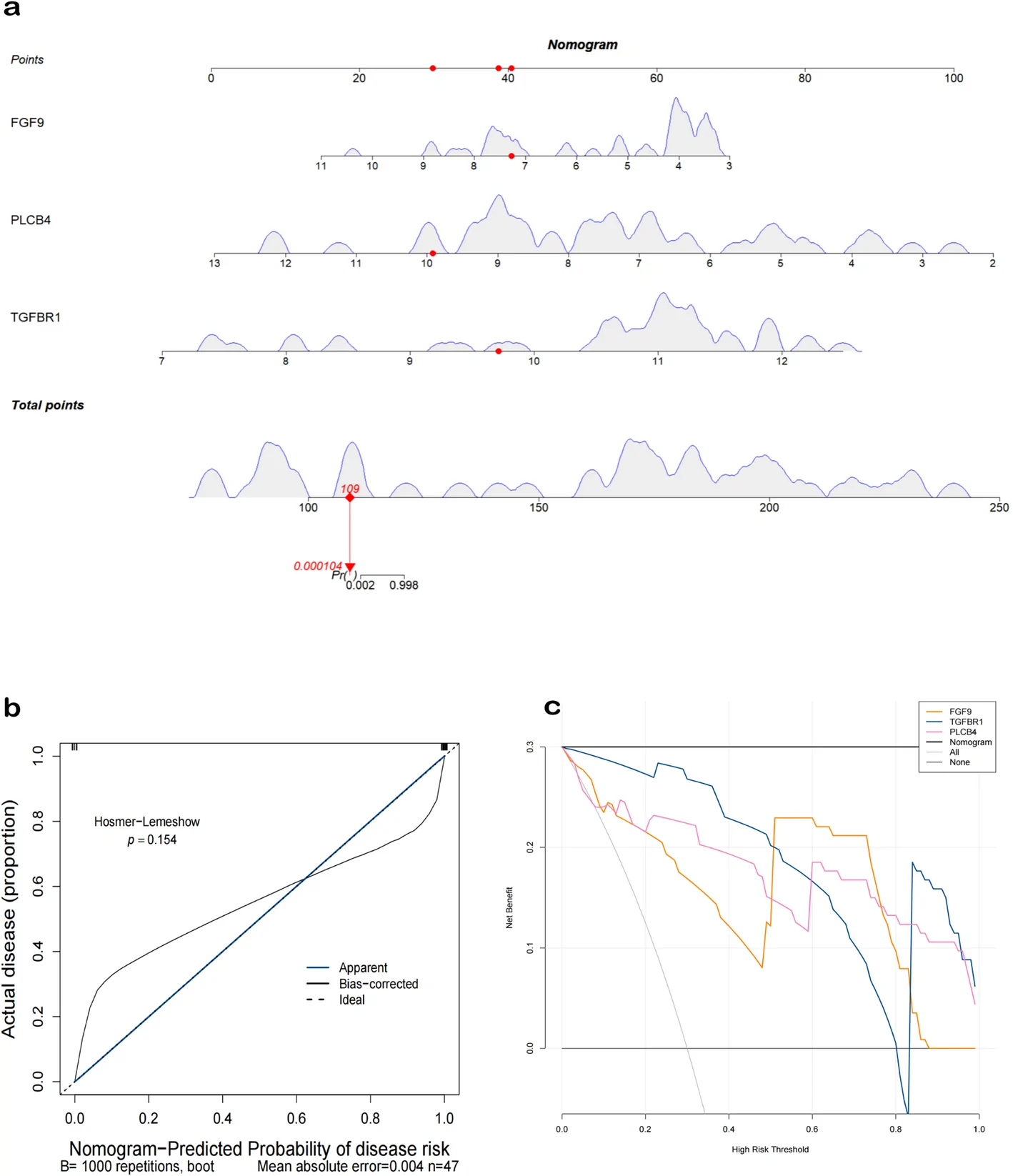
Evaluation of biomarker nomogram. (**a**) Biomarker-based nomogram for predicting GBM. (**b**) Calibration curve of the nomogram. (**c**) DCA of the nomogram

**Fig. 5:**
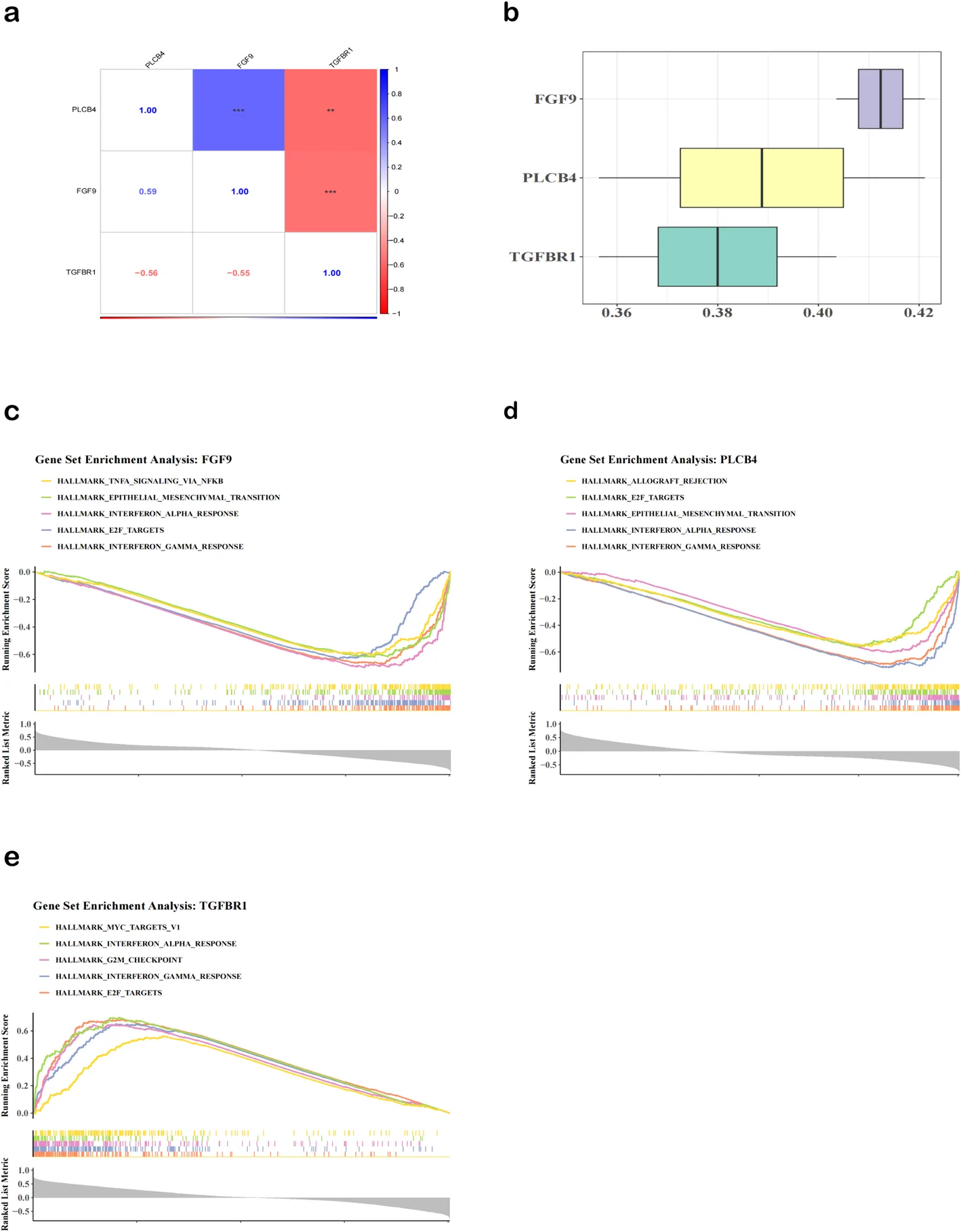
Biomarkers correlation network. **a** Spearman’s rank correlation of biomarkers, Red: Positive correlations, Blue: Negative correlations. **b** Raincloud plot of biomarker functional similarity. **c**–**e** Gene set enrichment analysis: FGF9, PLCB4, TGFBR1

**Fig. 6:**
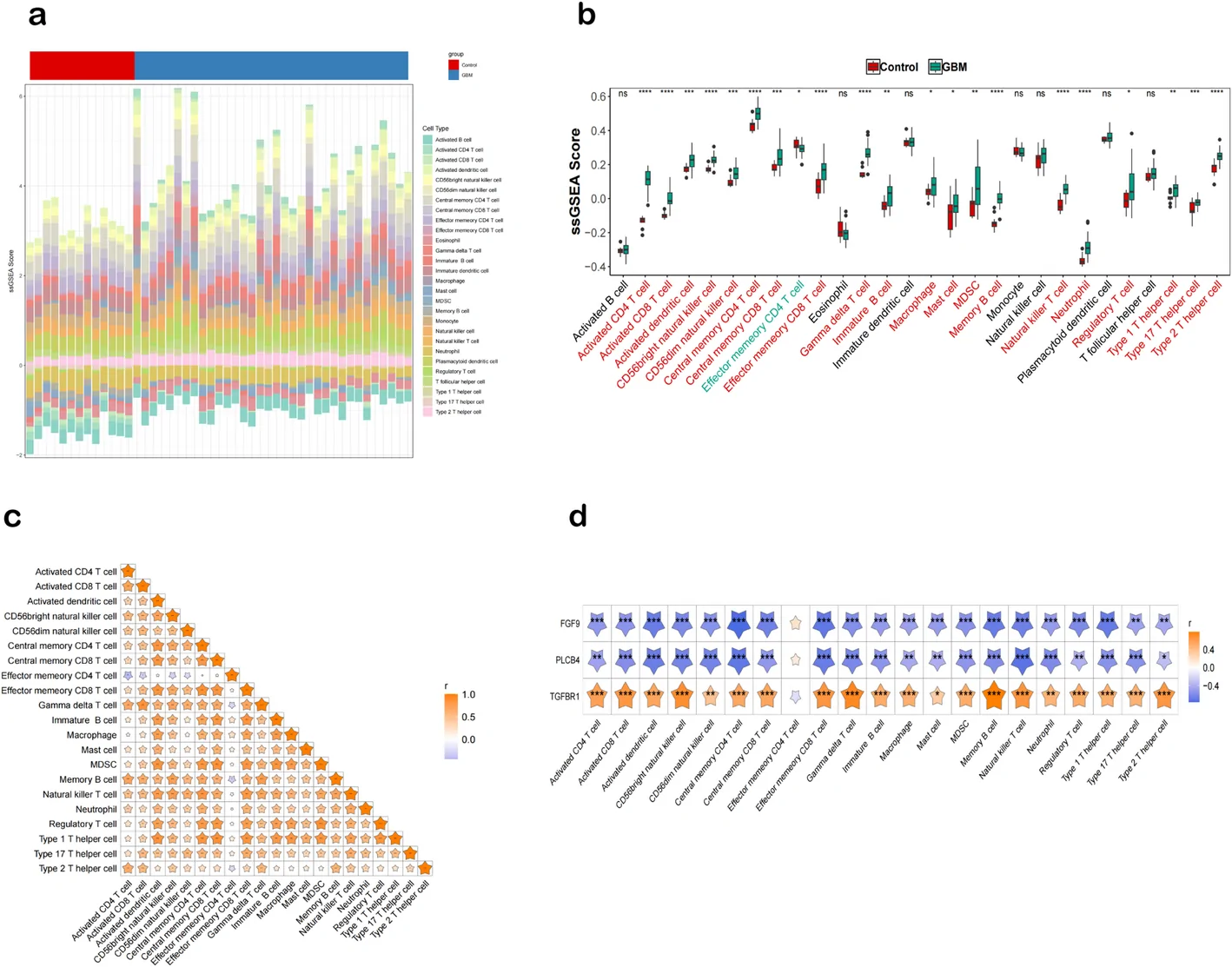
Immune infiltration analysis. (**a**) Stacked bar plot showing the relative proportions of 22 immune cell types across samples. (**b**) Differential immune cell infiltration in GBM vs. Control. (**c**) Immune cell correlation network. (**d**) Correlation heatmap between biomarkers and differentially infiltrated immune cells

**Fig. 7:**
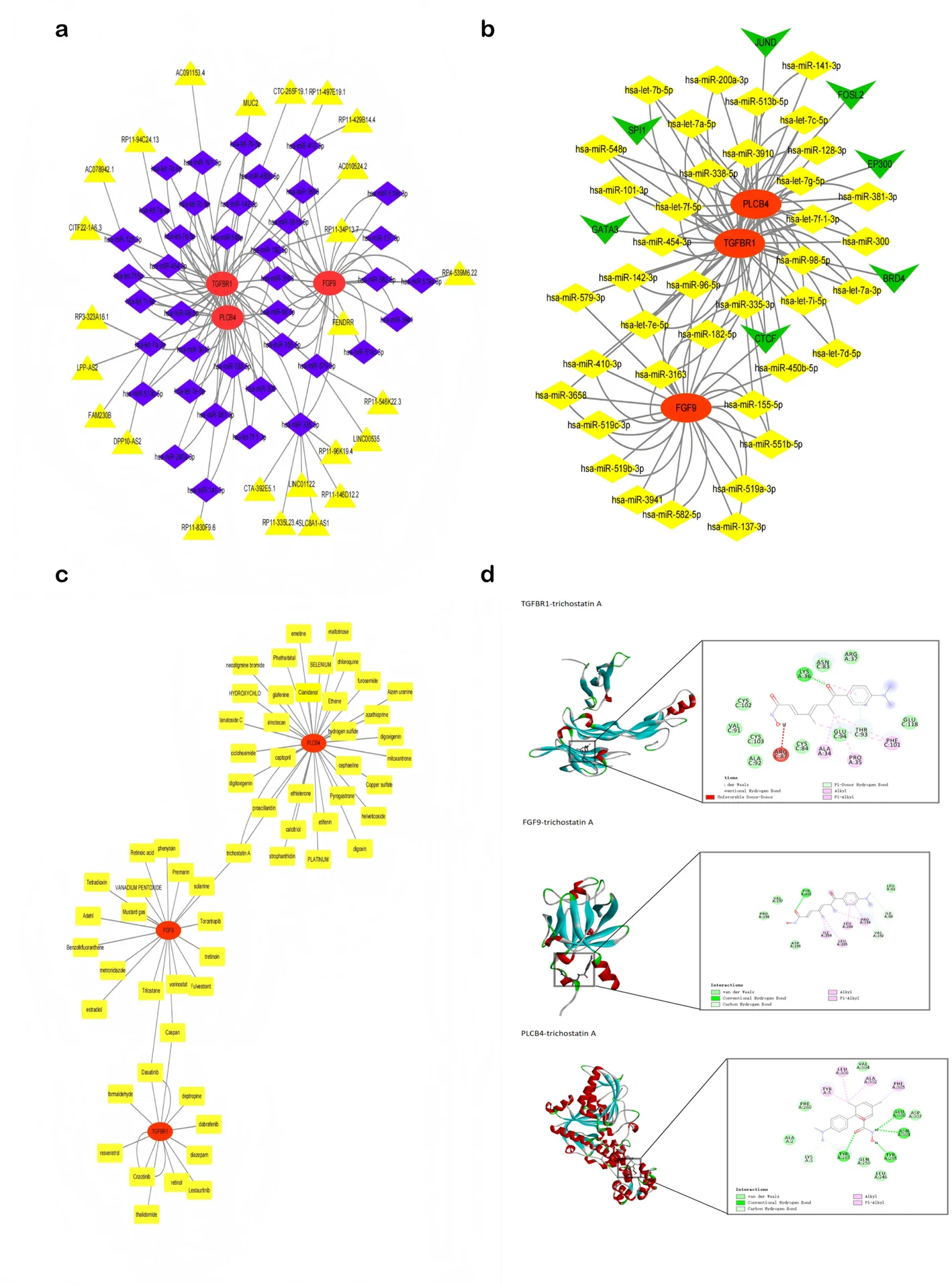
Multiple factors and drug effects on biomarker-centered networks. (**a**) lncRNA-miRNA-mRNA Regulatory Network, Red: Key genes, Yellow: lncRNAs, Blue: miRNAs. (**b**) TF-mRNA-miRNA regulatory network, Red: Key genes, Yellow: miRNAs, Green TF. (**c**) Biomarker-Drug Interaction Network, Red: Key genes, Yellow: Drug Candidates. (**d**) Molecular docking interaction diagram (TGFBR1, FGF9, PLCB4)

**Fig. 8:**
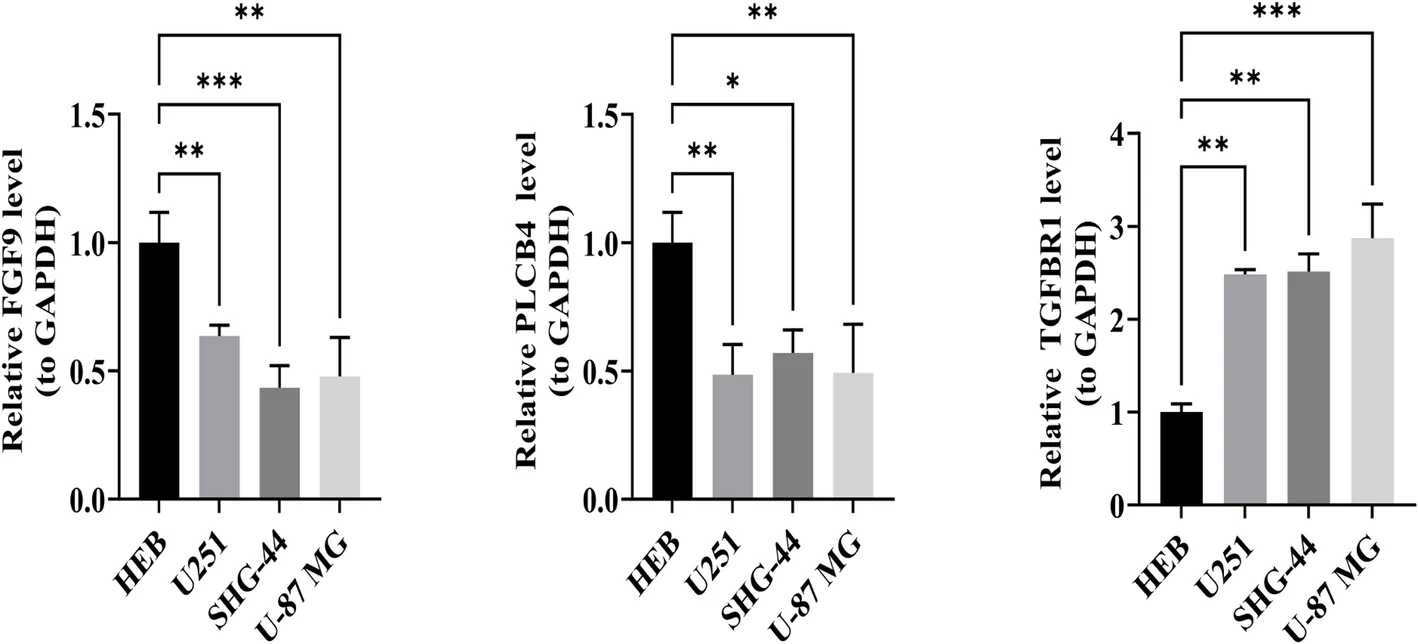
Relative level to GAPDH (FGF9, PLCB4, TGFBR1)

Furthermore, these candidate genes demonstrated significant enrichment in 31 Kyoto encyclopedia of genes and genomes (KEGG) signaling pathways, including but not limited to the Mitogen-activated protein kinase (MAPK) signaling pathway (*P* < 0.05) (Fig. 1e, Table S4). Afterwards, the protein-protein interaction (PPI) network, consisting of 10 nodes and 16 edges, was constructed (Fig. 1f). Moreover, maximal clique centrality (MCC) was utilized to screen the interacting genes, which were then classified according to the algorithm scores. The top 10 genes of the algorithm were visualized, which demonstrated robust interactions among genes including SFN, CHEK2, EPCAM, FGFR3, POSTN, ITGB1, TGFBR1, GSTP1, FGFR2, and FGF9 (Fig. 1g).

### FGF9, TGFBR1, and PLCB4 were identified as biomarkers for pGBM

Machine learning algorithms were applied to refine candidate genes. LASSO regression identified five feature genes, while SVM-RFE selected ten genes. Overlap confirmed three biomarkers: FGF9, TGFBR1, and PLCB4 (Fig. 2e), which were prioritized for further analysis due to their consistent expression trends. ROC curves showed AUC > 0.7 in both datasets (Fig. 3a, b), and expression analysis confirmed FGF9 and PLCB4 were down-regulated, while TGFBR1 was up-regulated in pGBM (*P* < 0.05; Fig. 3c, d). Chromosomal localization placed FGF9, TGFBR1, and PLCB4 on chromosomes 13, 9, and 20, respectively (Fig. 3e), supporting their genetic relevance. Sub-cellular localization analysis revealed that the gene-expressed products of the three genes mainly localized in the nucleus (Fig. 3f-h), suggesting their potential involvement in gene expression regulation processes.

### A nomogram that could effectively predict the risk of pGBM was constructed

A nomogram model was rebuilt to evaluate the combined ability of FGF9, TGFBR1, and PLCB4 to predict the risk of pGBM (Fig. 4a). The slope of the calibration curve was greater than 0.5 and close to the reference line, suggesting a better agreement between the predicted and actual probabilities (Fig. 4b). Moreover, the decision curve analysis (DCA) results demonstrated that the net benefit rate of the nomogram model was greater than that of a single biomarker, indicating its superior predictive performance (Fig. 4c). These results suggested that the nomogram model had good predictive ability, as supported by the calibration and decision curve analyses.

### Biomarkers shared enriched signaling pathways

Correlation analysis revealed that PLCB4 was positively correlated with FGF9 (cor = 0.59), negatively correlated with TGFBR1 (cor = − 0.56), and there was also a negative correlation between FGF9 and TGFBR1 (cor = -0.55), with all these correlations being significant (|cor| > 0.3, *P* < 0.05) (Fig. 5a). Additionally, functional similarity analysis demonstrated that the functional similarities between FGF9 with PLCB4 and TGFBR1, PLCB4 with TGFBR1 and FGF9, and TGFBR1 with FGF9 and PLCB4 were 0.412, 0.389, and 0.380, respectively (Fig. 5b). Furthermore, Gene set enrichment analysis (GSEA) outcomes indicated that among the top five markedly enriched signaling pathways of FGF9, TGFBR1, and PLCB4, the commonly enriched pathways were those related to INFγ response, INFα response, and E2F target genes (False Discovery Rate [FDR] < 0.05) (Fig. 5c-e). These findings suggested that these three biomarkers might have impacted the progression of pGBM by influencing the functions of these signaling pathways, particularly in immune response [17] and cell cycle regulation [18], which aligns with their proposed roles as key regulators.

### Biomarkers were implicated in the immune microenvironment of pGBM

The infiltration abundances of 28 immune cells between the pGBM and control groups in the GSE50161 dataset were demonstrated using a stacked-bar plot (Fig. 6a). A total of 21 differentially infiltrated immune cell subsets (DICs) were identified between the two groups, including activated CD4 T cell and others (Fig. 6b). Among these DICs, the infiltration score of effector memory CD4 T cells was lower in the control group, while the opposite was true for the other DICs (*P* < 0.05).

Correlation analysis exhibited that regulatory T cell and myeloid-derived suppressor cells (MDSC) exhibited the most robust positive correlation, while activated CD4 T cell and effector memory CD4 T cell exhibited the most robust negative correlation (cor= − 0.43, *P* = 3.64 × 10^− 3^) (Fig. 6c, Tables S5-S6). Additionally, relying on the screening criteria of |cor|>0.3 and *P* < 0.05, 20 DICs were markedly correlated with the three biomarkers. Specifically, FGF9 had the most negative correlation with central memory CD4 T cell (cor= − 0.76, *P* = 5.89 × 10^− 10^), PLCB4 also had the most negative correlation with central memory CD4 T cell (cor= − 0.70, *P* = 5.24 × 10^− 8^), and TGFBR1 had the most positive correlation with memory B cell (cor = 0.78, *P* = 1.16 × 10^− 4^) (Fig. 6d, Tables S7-S8). These findings suggested that the biomarkers might influence pGBM by affecting the functions of immune cells, thus supporting their role in pGBM pathogenesis.

### Multiple factors and drug effects on biomarker-centered networks

As depicted in (Fig. 7a), a lncRNA-microRNA (miRNA)-mRNA regulatory network was constructed, which included three biomarkers, 39 miRNAs, and 25 long non-coding RNAs (lncRNAs). For instance, RP11-34 PB.7 regulated the expression of FGF9 and TGFBR1 through hsa-miR-137-3p and hsa-miR-182-5p, respectively. LPP-AS2 regulated the expression of TGFBR1 via hsa-miR-7a-3p. Meanwhile, a TF-mRNA-miRNA regulatory network was built (Fig. 7b), containing three biomarkers, 45 messenger mRNAs, and 7 transcription factors (TFs). Among them, JUND, FOSL2, EP300, and CTCF targeted PLCB4. In addition, drug prediction results indicated that trichostatin targeted both PLCB4 and FGF9, while basatinib and caspan simultaneously targeted TGFBR1 and FGF9 (Fig. 7c). Furthermore, the results of molecular docking exhibited that the binding energy between FGF9, PLCB4, TGFBR1 and trichostatin A was − 6.3, − 7.6 and − 7.9 kcal/mol (Fig. 7d; Table 1). The in silico data showed that the binding of these three drugs to the biomarkers was relatively stable based on docking scores. These preliminary findings imply that the drugs could have potential therapeutic effects on pGBM, but experimental studies are needed to confirm this.

**Table 1:** Molecular docking of trichostatin A with biomarkers related to pGBM

Protein	Drug	Docking score (kcal/mol)
FGF9	Trichostatin A	− 6.3
PLCB4	Trichostatin A	− 7.6
TGFBR1	Trichostatin A	− 7.9

### Biomarkers expression

Reverse transcription quantitative polymerase chain reaction (RT-qPCR) was used to evaluate the expression levels of biomarkers in HEB, U251, SHG-44 and U-87 MG groups. When compared with the HEB group, the U251, SHG-44, and U-87 MG groups all exhibited high expression of TGFBR1 and low expression of PLCB4 and FGF9, and the differences were statistically significant (*P* < 0.05) (Fig. 8).

## Discussion

pGBM is a highly aggressive pediatric CNS malignancy. Studies suggest that Exo may play important roles in pGBM progression, as research has identified key miRNA networks in Exo that regulate long-term survival genes in pGBM patients. The investigation of Exo in pGBM may provide new treatment opportunities absent at the time of initial diagnosis. In this study, 34 candidate genes were screened out through cross-screening with endoplasmic reticulum-related genes. These candidate genes were markedly enriched in various biological functions and signaling pathways. A PPI network was then constructed, and 10 key interacting genes were screened out. Finally, FGF9, TGFBR1, and PLCB4 were identified as biomarkers for pGBM. The constructed nomogram model exhibited good predictive performance. Correlation analysis indicated that these biomarkers were closely related to immune cell infiltration and had significant associations with each other. In addition, the study also constructed lncRNA-miRNA-mRNA and TF-mRNA-miRNA regulatory networks, and predicted that drugs such as trichostatin could target these biomarkers. Molecular docking exhibited that the drugs bound stably to the biomarkers, suggesting their potential therapeutic value for pGBM.

This study identified 34 candidate genes through systematic screening, which may participate in the malignant transformation of pGBM via endoplasmic reticulum stress-related pathways. Functional enrichment analysis revealed that these candidate genes were markedly enriched in GO terms, including regulation of cell division, apical junction complex, and transmembrane receptor protein kinase activity. Among these, dysregulation of cell division may contribute to uncontrolled tumor cell proliferation, while functional impairment of the apical junction complex could be associated with reduced intercellular adhesion and enhanced invasiveness of tumor cells [19–21]. Alterations in transmembrane receptor protein kinase activity may persistently activate pro-oncogenic signaling by modulating growth factor pathways, such as the receptor tyrosine kinase (RTK) cascade [22, 23]. Candidate genes are significantly enriched in signaling pathways such as MAPK. The MAPK pathway plays a critical role in the pathogenesis and progression of glioma, regulating multiple malignant processes including tumor cell proliferation, invasion, immune evasion, angiogenesis, and chemotherapy resistance. Thus targeting p38 MAPK is expected to improve patient prognosis for glioblastoma (GBM) and decrease the chance of GBM tumor recurrence [24]. These discoveries establish a crucial basis for a more profound elucidation of the pathogenesis of glioma and foster the advancement of innovative targeted therapeutic strategies.

This study identified FGF9, TGFBR1, and PLCB4 as potential biomarkers for pGBM. FGF9, a member of the FGF family, exhibiting broad mitogenic and cell survival activities while participating in various biological processes such as embryonic development and cell growth [25–27]. Notably, FGF9 is widely expressed in the CNS, and its deficiency in cerebellar purkinje cells leads to impaired cerebellar development [28]. Medulloblastoma, one of the most common malignant brain tumors in children, may involve FGF9 in promoting tumor cell proliferation and survival, with high FGF9 expression correlating with tumor invasiveness and chemoresistance in this disease [29]. Studies have demonstrated that FGF9 can stimulate the growth of glioma and meningioma cells, while some human meningioma cells are capable of secreting FGF9. Importantly, these FGF9 secreting meningioma cells also secrete basic fibroblast growth factor, suggesting that FGF9 may function as an autocrine growth stimulator in certain meningiomas and serve as a component of the autocrine signaling network in these tumors [30]. Although a paucity of direct studies on FGF9 in pGBM exists, its potential significance and therapeutic value in childhood brain tumors have garnered increasing attention. Future research is expected to further elucidate the specific mechanisms and clinical applications of FGF9 in pGBM. Exhibited down regulation, which may impair its tumor-suppressive role in cell proliferation regulation and contribute to aberrant angiogenesis in the TME [31, 32]. Members of the TGF-β family, including the TGFBR1 mediated signaling pathway, play crucial roles in various biological processes of multiple cancers. TGF-β activates downstream sma and mad related protein (SMAD) pathways through TGFBR1, promoting stemness, immunosuppression, and therapy resistance in tumor cells [33–35]. Under hypoxic conditions, glioma stem cells activate TGF-β signaling via autocrine TGFBI (TGF-β-induced protein), which further enhances TGFBR1 mediated maintenance of stemness and invasive capacity [36]. The TGFB1/TGFBR1 axis constructs a profoundly immunosuppressive microenvironment by driving the epithelial-mesenchymal transition (EMT) phenotype, promoting the recruitment and accumulation of M2-type macrophages, and exhausting CD8 + T cells. The combined application of TGFB1/TGFBR1 and PD-1/PD-L1 inhibitors shows promise as a novel therapeutic strategy with low-grade gliomas [37]. In high-grade gliomas, the TGF-β signaling pathway constitutes a tumor-promoting network that is formed in different ways involving different receptors and their downstream effectors. ALK1/Smad1/5 enhances endothelialization and tumor spread, whereas ALK5/Smad2/3 and ALK7 induce the epithelial-mesenchymal transition, stemness, and therapy resistance. Therefore, therapies against these pathways are predicted to simultaneously restrain tumor progression. While concurrently demonstrating the therapeutic potential of TGFBR1 inhibitors against this malignancy. This dual-targeting approach enables multidimensional suppression of glioma, positioning it as a highly promising therapeutic direction [38, 39]. This study revealed that TGFBR1 is significantly upregulated in pGBM, indicating its critical role in tumor pathogenesis. Studies show that hyperoxic stimuli like radiotherapy upregulate expression of PLCB1 which activates the PLC/PKC/NF-κB signaling pathway. This series of events enhances the survival of cells (chemoresistance) and activates a mesenchymal gene program, causing temozolomide resistance and an increase in glioma invasiveness [40]. It remains an open question whether PLCB4, another member of the PLC-β family, regulates pGBM aggressiveness and therapy resistance. PLCB4 expression was low in pGBM, indicating that different members of the same protein family may have different functions. In colorectal cancer, miR-1271-5p-mediation suppression of PLCB4 may activate MAPK and VEGF signalling, contributing to drug resistance, this suggests the potential therapeutic relevance of the miR-1271-5p/PLCB4 axis [41]. This note is consistent with our results that together support a link between low expression of PLCB4 with tumourigenesis and treatment resistance. Hence, further studies should investigate the expression profile and function of PLCB4 in pGBM.

The study data analysis established FGF9, TGFBR1, and PLCB4 as the relevant prognostic markers. A nomogram using these markers performed better than the single marker approach with regards to predictive accuracy. This nomogram showed good risk stratification ability. Validation with calibration curves and DCA showed excellent predictive capacity. The markers found on chromosomes 13 (FGF9), 9 (TGFBR1) and, 20 (PLCB4) are located in genomic regions that are often affected by copy number variations (CNVs) and epigenetic dysregulation in GBM, suggesting that their dysregulation may be linked to genomic instability [42].

Correlation analysis revealed a significant positive association between PLCB4 and FGF9 expression, whereas TGFBR1 showed an inverse correlation with both genes, implying a sophisticated regulatory network among the three biomarkers. Subsequent functional similarity analysis further identified moderate similarity scores for both the FGF9-PLCB4 (0.412) and FGF9-TGFBR1 (0.389) pairs. These findings suggest these genes may act synergistically or antagonistically via shared pathways during oncogenesis.

GSEA results showed that these biomarkers were commonly enriched with genes related to the following pathways: INF-γ response, INF-α response, and E2F target genes. These pathways play crucial roles in cell proliferation, immune response, and cell cycle regulation. Existing studies have shown that interfering with related signaling pathways may regulate antigen presentation and immune cell aggregation, thereby affecting the TME and providing new ideas for tumor immunotherapy [43, 44]. E2F target genes are closely associated with invasive cell cycle, DNA repair, STAT3, TGFβ, and WNT pathways, thereby affecting glioma formation. By regulating their expression, tumor prognosis can also be altered [45, 46]. These findings suggest that FGF9, TGFBR1, and PLCB4 may synergistically regulate these interconnected pathways, forming a network effect in the pathogenesis of pGBM, rather than functioning independently.

Immunoinfiltration analysis revealed 21 differentially abundant immune cell populations between the pGBM group and control group. Among these, the infiltration score of effector memory CD4 + T cells was significantly lower in the control group, while regulatory T cells (Tregs) showed a strong positive correlation with MDSCs (cor = 0.87). Studies have confirmed that both the quantity, activity, and immunosuppressive function of Tregs and MDSCs were significantly upregulated in the GBM microenvironment, and they exhibited synergistic effects during anti-tumor immunosuppression [47, 48] Further correlation analysis found a negative correlation between activated CD4 + T cells and effector memory CD4 + T cells, FGF9 and PLCB4 show strong negative correlations with central memory CD4 + T cells, suggesting that their down-regulation may impair memory T cell infiltration/function, thereby affecting long-term anti-tumor immunity [49]. While upregulation of transforming growth factor beta receptor 1 (TGFBR1) positively correlated with memory B cells. As a key receptor of the TGF-β signaling pathway, TGFBR1 may mediate the early inhibitory effect of TGF-β on tumor cell growth; on the other hand, during tumor progression, it is exploited by tumor cells to transmit immunosuppressive signals, inhibit the function of effector immune cells, and promote the expansion of immunosuppressive populations such as Tregs, thereby facilitating tumor immune escape [50, 51]. Downregulation of FGF9 and PLCB4 may dysregulate interferon signaling, reducing proinflammatory cytokine secretion and impairing effector T cell activation, thereby cooperatively facilitating immune escape [52]. These studies demonstrate how the identified biomarkers coordinate to form an immunosuppressive microenvironment, which in turn affects pGBM pathogenesis.

This study integrated multi-omics data with FGF9, TGFBR1, and PLCB4 as core biomarkers to construct a hierarchical regulatory network, suggesting their potential as therapeutic targets. Non-coding RNA regulatory layer, a lncRNA-miRNA-mRNA network comprising 25 lncRNAs, 39 miRNAs, and three biomarkers, suggests that non-coding RNAs may modulate post-transcriptional regulation via competitive endogenous RNA (ceRNA) mechanisms. This complex ceRNA network likely forms a post-transcriptional regulatory hub in pGBM, driving aberrant biomarker expression and tumor progression. TF Regulatory Network Seven TFs-including JUND, FOSL2, EP300, and CTCF-were identified as upstream regulators of PLCB4, primarily involved in cell proliferation, apoptosis, and signal transduction [53–56]. Specifically, JUND and FOSL2 (key components of the AP-1 complex) promote tumor cell invasion via downstream gene dysregulation [57, 58]. CTCF, a chromatin architectural protein, may be reshaping the 3D genome organization to regulate PLCB4 expression [59]. These results suggest that aberrant biomarker expression arises from cooperative interactions between transcription factor networks and non-coding RNAs, establishing multi-level regulatory nodes critical to pGBM pathogenesis.

Trichostatin A demonstrates robust binding to the three biomarkers, underscoring its therapeutic potential in pGBM [60]. Meanwhile, dual inhibition of TGFBR1 and FGF9 by bosutinib and caspase inhibitors highlights a strategic advantage in multi-target therapy, which may reduce the likelihood of drug resistance via synergistic effects [61]. Inhibition of TGFBR1 may shift the immune landscape by suppressing the recruitment of regulatory T cells and MDSCs, which contributes to alleviating immunosuppression and boosting anti-tumor immunity [62]. Inhibiting FGF9 might disrupt angiogenesis caused by FGF signals, providing dual therapeutic effects. The FGF9/TGFBR1/PLCB4 connection plays a multi-layer regulatory role in glioblastoma and provides hints for targeted therapies that may exploit epigenetic, transcriptional and immune microenvironment vulnerabilities.

Multi-omics and immune contexture analysis pinpoint FGF9, TGFBR1, and PLCB4 as critical biomarkers and potential therapeutic nodes in pGBM progression. Multi-omics analysis highlights FGF9, TGFBR1, and PLCB4 as critical biomarkers, but this study is limited by its reliance on bioinformatics data and mRNA-level validation. Future research should focus on multiple aspects to enhance the translational value of this study: First, causal confirmation through protein expression validation (e.g., Western blot) and functional experiments (e.g., gene knockdown) in patient-specific pGBM models; second, integrate multi-omics data and epigenomic analysis to deeply explore the interactions among the FGF9/TGFBR1/PLCB4 network, exosomes, and the immune microenvironment; additionally, develop clinical diagnostic tools (e.g., liquid biopsy) and targeted therapies (e.g., multi-inhibitor combinations), and promote precision medicine through interdisciplinary collaboration, thereby translating bioinformatics findings into practical clinical applications.

## Methods and materials

### Data acquisition

The GEO database (http://www.ncbi.nlm.nih.gov/geo/) was utilized to obtain the pGBM related datasets (GSE50161 and GSE35493). GSE50161 dataset, with the platform of GPL570, was utilized as a training set, and it contained 34 tumor tissue samples from pGBM patients and 14 control samples [63]. Meanwhile, the GSE35493 dataset, which was obtained relying on the GPL570 platform, was used as a validation set. It comprised 12 tumor tissue samples from pGBM patients and 9 control samples [64]. A total of 121 ERGs were obtained from ExoBCD [65].

### Expression analysis

To select DEGs, the sorting criteria were *P* < 0.05 and |log_2_ FC| >1 (GSE50161). Using ggplot2 (v 3.4.1) [66] and pheatmap packages (v 1.0.12) [67], a volcano plot and a heat map were generated to represent the expression of DEGs, respectively, labeling the top 10 up-regulated and down-regulated DEGs in the volcano plot.

### Discovery and enrichment examination of potential candidate genes

The VennDiagram package (v 0.1.9) [68] was utilized to cross DEGs and ERGs to obtain candidate genes. The clusterProfiler package (v 4.2.2) [69]was leveraged to conduct GO and KEGG enrichment analyses on candidate genes (adj. *P* < 0.05), aiming to gain insights into the associated biological functions and pathways. For visualization, the top 30 significant terms were selected. Specifically, GO biological functions were composed of CC, MF, and BP. After that, these candidate genes were imported into the Search Tool for the Retrieval of Interacting Genes/Proteins (STRING) database (http://www.string-db.org/), with the species set as ‘Homo sapiens’ and an interaction score threshold of > 0.4 for constructing the PPI network. Besides, the visualization of this network was performed using Cytoscape software (v 3.8.2) [70]. Further, MCC of the plug-in CytoHubba in Cytoscape software was utilized to screen the interacting genes, which were then classified according to the algorithm scores, and the top 10 genes of the algorithm were visualized.

### Recognition of biomarkers

For feature gene selection, the LASSO regression analysis was carried out using the glmnet package (v 4.1.8) [71]. In contrast, the SVM-RFE algorithm analysis was executed via the e1071 package (v 1.7.13) [72]. Importantly, 10-fold cross-validation was used in the LASSO analysis. Immediately thereafter, feature genes from the two algorithms were crossed to provide candidate biomarkers. Furthermore, Receiver operating characteristic (ROC) curve analysis using the pROC package (v 1.18.0) [73] and expression evaluation of candidate biomarkers were performed in two datasets. Candidate biomarkers with the AUC greater than 0.7, consistent expression trends, and significant differences between pGBM and control groups in both GSE50161 and GSE35493 datasets were selected as biomarkers.

### Evaluation of the predictive ability of biomarkers for pGBM

To determine whether the recognized biomarkers were reliable for predicting pGBM patients, rms package (v 1.7–14) [74] was leveraged to build a nomogram model of biomarkers in GSE50161 set. Moreover, calibration curves and DCA were adopted to evaluate the accuracy of the predictive capability of the nomogram model. By the way, DCA was implemented employing the ggDCA package (v 1.2).

### Functional enrichment analyses

In all samples of the training set GSE50161, Spearman correlation analysis was performed among the biomarkers using the R package “psych” (|cor|) > 0.3, *P* < 0.05 (v 2.1.6) [75]. Meanwhile, the R package “GOSemSim” (v 2.26.1) [76] was utilized to calculate the GO semantic similarity of biomarkers. Specifically, the geometric means of biomarkers at three levels, BP, MF, and CC, were computed to obtain the final scores. These scores were then used to evaluate the functions of biomarkers at the three GO levels. Moreover, GSEA was implemented utilizing the clusterProfiler package (v 4.2.2) to comprehend the biological functions of biomarkers engaged in the development of pGBM [69]. First, Spearman correlation coefficients were computed for each biomarker and all genes separately in all samples from the GSE50161 dataset. Subsequently, the genes were classified from largest to smallest relying on the correlation coefficients to obtain the ranked list of genes corresponding to each biomarker. Immediately thereafter, GSEA was conducted relying on h.hallmark.v7.4.symbols.gmt in the Molecular Signatures Database (MSigDB) as the reference gene set [FDR < 0.05]. The top five signaling pathways (classified by enrichment score) of the biomarker enrichment results were visualized using the enrichplot package (v 1.18.3) [77].

### Immune infiltration analysis

The infiltration scores for 28 immune cells were computed using the “ssGSEA” package. Subsequently, the samples were filtered according to the confidence level of the samples, followed by comparing the differences in the infiltration scores of these immune cells between groups using the Wilcoxon test to obtain DICs (*P* < 0.05). Immediately following this, Spearman correlation analysis was applied via the psych package (v 2.1.6) [75] to investigate the potential relationships among DICs, as well as between biomarkers and DICs (|cor| > 0.3, *P* < 0.05).

### Creation of regulatory networks

We leveraged the “multiMiR” package (v 1.24.0) [78] to forecast miRNAs functioning as regulatory modulators of messenger RNAs(mRNAs), thereby generating comprehensive miRNA-mRNA regulatory interaction datasets. The SponGECan database (https://spongescan.readthedocs.io/en/latest/Home/) was applied to predict lncRNAs that manifested regulatory associations with these recognized miRNAs. A comprehensive lncRNA-miRNA-mRNA and miRNA-lncRNA relationship pair was built. After that, biomarkers’ TFs were predicted using KnockTF database.

### Drug prediction and molecular docking

To explore drugs with potential therapeutic effects on biomarkers, the DSigDB database was leveraged to predict drugs targeting biomarkers. The constructed biomarker-drug network was then visualized using Cytoscape software (v 3.8.2) [70]. Notably, this work selected drugs corresponding to each biomarker, and molecular docking analyses were conducted separately.

The protein crystal structures corresponding to each biomarker were downloaded from the Protein Data Bank (PDB) database, followed by obtaining the 3D structure of the active compound in the PubChem database. After that, AutoDock Tools software was utilized to predict the binding activity of proteins with compounds, and PyMOL software (v 3.3.0) was used for molecular docking and visualization of biomarkers and active components.

### RT-qPCR

The human cell lines (pGBM) used in this study, including HEB human glial cells, U251 (human glioma cells), SHG-44 (human glioma cell line), and U-87 MG (human brain astrocytoma cells), were obtained from American Type Culture Collection (ATCC) and were cultured under standard conditions. As all cell lines were established commercial models and no primary human tissues were directly involved, approval from an institutional ethics committee was not required. All experimental procedures were conducted in strict accordance with the principles of the Declaration of Helsinki and institutional biosafety guidelines. Following the manufacturer’s protocols, total RNA was extracted from 12 samples using TRIzol (Vazyme, China). Reverse transcription was performed using the HP All-in-one qRT Master Mix II RT203-Ver.1 kit (Kunming Yungeng Biotechnology Co., Ltd.). Subsequently, qPCR experiments were carried out. For precise evaluation of gene expression levels, normalization was conducted using the housekeeping gene GAPDH. The relative gene expression values were determined using the 2^-ΔΔCt^ method, as previously reported in literature (*P* < 0.05) [79].

### Statistical analysis

R software (v 4.2.2) was leveraged to handle and examine the data. The Wilcoxon test was applied to assess the disparities between groups. Statistical significance was established when the *P*-value was below 0.05.

## Supplementary Information

Below is the link to the electronic supplementary material.


Supplementary Material 1: Table S1. The table of differentially expressed genes (DEGs) between pediatric glioblastoma (pGBM) and control groups in the GSE50161 dataset.



Supplementary Material 2: Table S2. The list of 34 candidate genes in the study of pediatric glioblastoma (pGBM).



Supplementary Material 3: Table S3. The table of results of Gene Ontology (GO) enrichment analysis for 34 candidate genes in the study of pediatric glioblastoma (pGBM).



Supplementary Material 4: Table S4. The table of results of Kyoto Encyclopedia of Genes and Genomes (KEGG) pathway enrichment analysis for 34 candidate genes was presented.



Supplementary Material 5: Table S5. The matrix of Spearman correlation coefficients between differentially infiltrated immune cells (DICs).



Supplementary Material 6: Table S6. The matrix of P-values for correlations between differentially infiltrated immune cells (DICs).



Supplementary Material 7: Table S7. The matrix of Spearman correlation coefficients between biomarkers (FGF9, TGFBR1, PLCB4) and differentially infiltrated immune cells (DICs).



Supplementary Material 8: Table S8. The matrix of P-values for correlations between biomarkers (FGF9, TGFBR1, PLCB4) and differentially infiltrated immune cells (DICs).


## Data Availability

The datasets analyzed during the current study are publicly available. The transcriptomic data can be accessed from the Gene Expression Omnibus (GEO) database under accession numbers GSE50161 and GSE35493. The exosome-related gene list was obtained from the ExoBCD database (http://www.exobcd.liumwei.org/). Protein-protein interaction analysis was performed using the STRING database (version 12.0, https://string-db.org/). Gene set enrichment analysis employed the Hallmark gene sets (v7.4) from the Molecular Signatures Database (MSigDB) (https://www.gsea-msigdb.org/gsea/msigdb). Drug prediction was conducted using the DSigDB database (http://tanlab.ucdenver.edu/DSigDB). Molecular structures were retrieved from PubChem (https://pubchem.ncbi.nlm.nih.gov/) and the Protein Data Bank (PDB) (https://www.rcsb.org/). Regulatory network predictions utilized the SponGECan (https://spongescan.readthedocs.io/) and KnockTF (http://www.licpathway.net/KnockTF/) databases. All data supporting the findings of this study are available from the corresponding author upon reasonable request.

## Abbreviations

pGBM: Pediatric glioblastoma
CNS: Central nervous system
GEO: Gene expression omnibus
ERGs: Exosome-related genes
DEGs: Differentially expressed genes
ROC: Receiver operating characteristic
INF: Interferon
LncRNA: Long non-coding RNA
Exo: Exosomes
BBB: Blood-brain barrier
TME: Tumor microenvironment
GO: Gene Ontology
BP: Biological processes
CC: Cellular components
MF: Molecular functions
KEGG: Kyoto encyclopedia of genes and genomes
MAPK: Mitogen-activated protein kinase
PPI: Protein-protein interaction
MCC: Maximal clique centrality
LASSO: Least absolute shrinkage and selection operator
SVM-RFE: Support vector machine-recursive feature elimination
AUC: Area under the curve
DCA: Decision curve analysis
GSEA: Gene set enrichment analysis
FDR: False Discovery Rate
DIC: Differentially infiltrated immune cell subsets
MDSC: Myeloid-derived suppressor cells
miRNA: microRNA
ceRNA: competitive endogenous RNA
TF: Transcription factor
RT-qPCR: Reverse transcription quantitative polymerase chain reaction
RTK: Tyrosine kinase
SMAD: Sma and mad related protein
EMT: Epithelial-mesenchymal transition
CNVs: Copy number variations
Tregs: Regulatory T cells
PDB: Protein data bank
